# A Split Biotin Ligase Approach to Revealing Proteins Associated with Oligomeric Alpha-Synuclein During Aggregation

**DOI:** 10.21203/rs.3.rs-7697442/v1

**Published:** 2025-10-17

**Authors:** Analiese R. Fernandes, Abigail P. Owen, Ayman H. Faroqi, Jannifer Lee, Gunveen S. Sachdeva, Dmytro Morderer, Cody Hoffmann, Benjamin Madden, Shuwen Zhang, Yingxue Ren, Suelen L. Boschen, Akhilesh Pandey, Wilfried Rossoll, Pamela J. McLean

**Affiliations:** Mayo Clinic Graduate School of Biomedical Sciences; Mayo Clinic Office for Non-Clinical Education Programs; Mayo Clinic Graduate School of Biomedical Sciences; Mayo Clinic Department of Neuroscience; Mayo Clinic; Mayo Clinic Department of Neuroscience; Mayo Clinic; Mayo Clinic; Mayo Clinic; Mayo Clinic Department of Quantitative Health Sciences; Mayo Clinic Department of Neuroscience; Mayo Clinic; Mayo Clinic Department of Neuroscience; Mayo Clinic Department of Neuroscience

**Keywords:** alpha-synuclein, Lewy pathology, Lewy body disease, proximity proteomics, neurodegeneration, proteinopathy

## Abstract

Lewy pathology can form over decades in patients with Lewy body diseases, but the causal cellular mechanisms associated with this process remain unclear. This project aims to discover proteins that associate with monomeric and/or oligomeric alpha-synuclein during early stages of the aggregation process. To mimic the aggregation processes, cells expressing a synuclein-biotin ligase fusion protein were treated human recombinant pre-formed fibrils and subjected to BioSITe and mass spectrometry. Using a novel split biotin ligase fused to alpha-synuclein facilitated the identification of proteins specifically associated with multimeric alpha-synuclein. A total of 581 proteins were differentiated into potential interactors of monomeric versus multimeric alpha-synuclein in physiological versus aggregated conditions. The data reveal important phosphorylation mechanisms, connections to insulin processing, and a potential interaction with ALS-associated FUS. Interestingly, we identified that loss of specific interactions may contribute to pathology in patients with sporadic onset of Lewy body diseases. Future studies will validate both true interaction of highlighted proteins with alpha-synuclein, and the impact of such proteins on alpha-synuclein aggregation.

## Introduction

The primarily neuronal protein alpha-synuclein (aSYN) is implicated in neurodegeneration via its genetic association with Parkinson’s disease (PD) [1, 2] and its accumulation in Lewy body (LB) and Lewy neurite pathological inclusions [3]. Lewy pathology is found in numerous vulnerable brain cell populations across a spectrum of synucleinopathies, a group of diseases which includes PD and dementia with Lewy bodies. Additionally, aSYN pathology is not confined to synucleinopathies. Upwards of 40% of Alzheimer’s disease brains are found to have significant levels of Lewy pathology at postmortem evaluation [4], and incidental Lewy pathology is identified in 8–17% of non-disease, healthy, aged individuals at autopsy [5–10]. The significance of Lewy pathology is a hotly debated topic, with some perceiving LB formation to be a neuroprotective mechanism employed by the cell to sequester toxic aSYN species, while others consider LBs as the entity with a chokehold on the cell that sends it spiraling towards certain death. Regardless of the functional significance, LBs have the potential to offer insight into disease pathogenesis.

While recent studies have established that LBs contain a multitude of proteins [11–15], lipid membranes, and organellar fragments [16], their significance to disease pathogenesis remains elusive. Furthermore, Lewy pathology is only confirmed post-mortem even though PD patients can experience symptoms up to 30 years prior to clinical diagnosis [17]. A handful of studies have applied proteomics of LBs to glean insight into pathogenic disease mechanisms [11, 13, 14, 18]. While these studies have shed light on broad cellular components interacting with Lewy pathology at end-stage disease, they cannot distinguish proteins relevant to disease pathogenesis.

The advent of proximity proteomics and specifically the ability to tag proximal proteins in living cellular systems, such as BioID, TurboID, and APEX labelling [19–23] have resulted in multiple studies to identify potential aSYN interactors. These studies differ by labelling method/time, species of aSYN studied, cell model, and whether aggregation was induced or not. In experiments where aggregation was not induced, proximity proteomics identified proteins associated with phosphorylated mimics of aSYN in yeast [24], A53T mutant aSYN in SH-SY5Y cells [25], E46K mutants aSYN in HEK293 cells[26], or wild-type aSYN in primary rat neurons [15]. A comparison of interactomes between wildtype tau, mutant tau, wildtype aSYN and mutant aSYN in human embryonic stem cell (hESC) derived neurons [27] revealed 45 shared interactors of tau and aSYN, but neither delineated between wildtype vs. mutant proteins, nor baseline and aggregated states.

Two studies queried the interactome of wildtype aSYN in HEK293 cells during disease-associated aggregation, employing either preformed fibrils generated from postmortem synucleinopathy patient samples [28] or light-induced oligomerization [29]. Of note, neither of these studies, nor those mentioned above, require aSYN oligomers to form for labelling to occur. To address this gap, we have developed a novel split-biotin labeling system (Split Syn-BirA*) where multimers of aSYN are required to form to enable enzymatic activity and biotin labelling. Such a system, never applied to aSYN to our knowledge, enables us to distinguish interactors of multimeric aSYN from interactors of monomeric or aggregating aSYN, providing novel information to the field.

Here, we applied proximity proteomics to identify potential protein interactors of monomeric and oligomeric alpha-synuclein *during the formation of disease-like aggregation* to identify how associations with aSYN change during the aggregation process. We identified 581 proteins with the potential to interact with monomeric or multimeric aSYN in unseeded versus pathogenic states. Taken together, the data confirm many of the potential interactors detected in similar studies, giving confidence in the study design, reveal phosphorylation-associated mechanisms in unseeded multimeric aSYN, highlight potential connections between multimeric aSYN and insulin processing, and identify a novel interaction of multimeric aSYN with FUS protein. Overall, the data support a hypothesis that loss of interactions drive aSYN aggregation in patients with sporadic PD onset.

## Results

**Figure 1.**
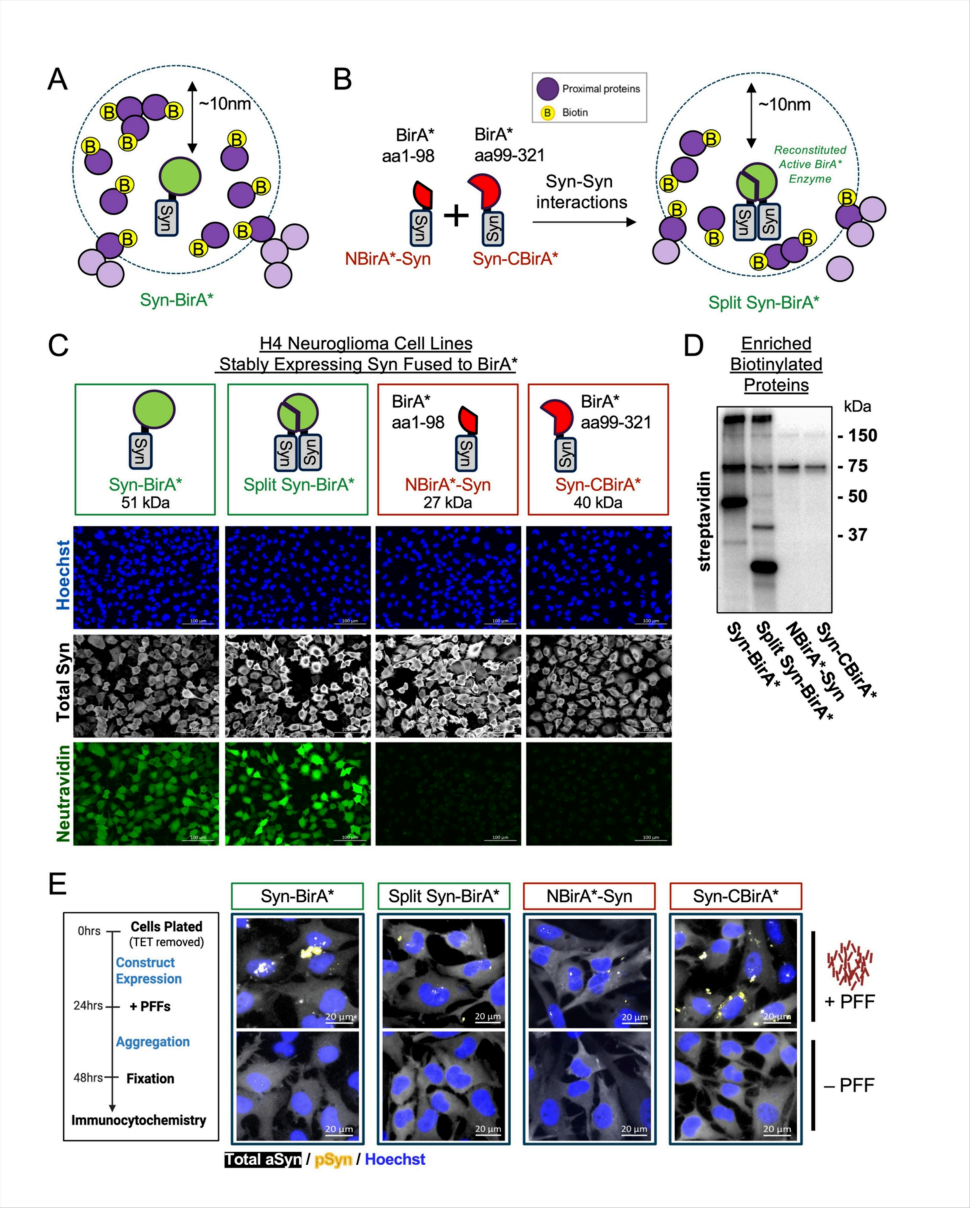
Development of aSYN-Fused BirA* Expressing Cell Lines **A.**Schematic highlighting the labelling radius (~10nm) of the full-length BirA* enzyme fused to alpha-synuclein (‘Syn-BirA*’). Proximal proteins are tagged with a biotin moiety in the presence of excess biotin. **B.** Inactive fragments of the BirA* enzyme are fused to alpha-synuclein (Syn) proteins. Syn-Syn interaction drives the complementation of BirA* fragments, reconstituting active biotin ligase enzymes (green) capable of biotinylating proximal proteins. **C.** H4 neuroglioma cell lines stably express: Syn-BirA*, NBirA*-Syn and Syn-CBirA* in the same cell (‘Split Syn-BirA*’), NBirA*-Syn only or Syn-CBirA* only. While all cell lines express Syn (grey-scale), only SynBirA* and Split Syn-BirA* demonstrate biotinylation of proteins, detected by neutravidin (green). **D.** Eluates from streptavidin-based enrichment of biotinylated proteins pulls out more proteins – including self-biotinylated Syn constructs – from Syn-BirA* and Split Syn-BirA* cell lysates. Bands present in NBirA*-Syn and Syn-CBirA* enrichment corresponds with known endogenously biotinylated proteins: pyruvate carboxylase (130kDa), 3-methylcrotonyl-CoA carboxylase (75kDa), and propionyl-CoA carboxylase (72kDa). **E**. Human recombinant pre-formed fibrils (PFFs) were used to mimic pathological aggregation. Syn phosphorylated at serine 129 (pSyn) is detected in cells exposed to PFFs (yellow), but not in cells treated with transfection reagent only (− PFF).

The goal of this study is to characterize changes in the interaction partners of monomeric and oligomeric alpha-synuclein (aSYN) under normal and pathological conditions. To do so we leveraged a biotin labelling approach, which is based on the fusion of a promiscuous mutant of *Escherichia coli* biotin ligase enzyme (BirA*) to aSYN. BirA* catalyzes the biotinylation of proximal proteins in the natural cellular environment [33], allowing for identification of potential protein interactors. To determine physiological and aggregate-specific interacting partners of aSYN, we developed a novel split-BirA* system (herein referred to as Split Syn-BirA*) whereby aSYN-aSYN interactions must occur to reconstitute a functional biotin ligase (Fig. 1B). Specifically, an aSYN protein N-terminally fused with BirA* amino acids 1–98 (herein referred to as NBirA*-Syn) must form at least a dimer with an aSYN protein C-terminally tagged with the BirA* amino acids 99–321 (herein referred to as Syn-CBirA*) to generate an enzymatically active biotin ligase.

We generated tetracycline-regulated stable cell lines expressing aSYN fused to the intact full-length BirA* (Syn-BirA* cells), co-expressing both NBirA*-Syn and Syn-CBirA* under the same bi-directional promoter (Split Syn-BirA* cells), or expressing only one construct, NBirA*-Syn or Syn-CBirA*. The resulting H4 neuroglioma cells stably overexpress aSYN but only demonstrate strong biotin ligase activity (detected by neutravidin) in the presence of a full or reconstituted BirA* (Fig. 1C and Supplementary Fig. 1). Many biotinylated proteins, including self-biotinylated aSYN, were captured in biotin-based enrichment from Syn-BirA* and Split Syn-BirA* cell lysates. Only endogenously biotinylated proteins and self-biotinylated proteins were enriched from negative control lysates (Fig. 1D). To model aSYN pathology, all cell lines were incubated with 1ug / mL human pre-formed fibrils (PFFs) to induce phospho-serine 129 aSYN positive aggregates. (Fig. 1E).

**Figure 2.**
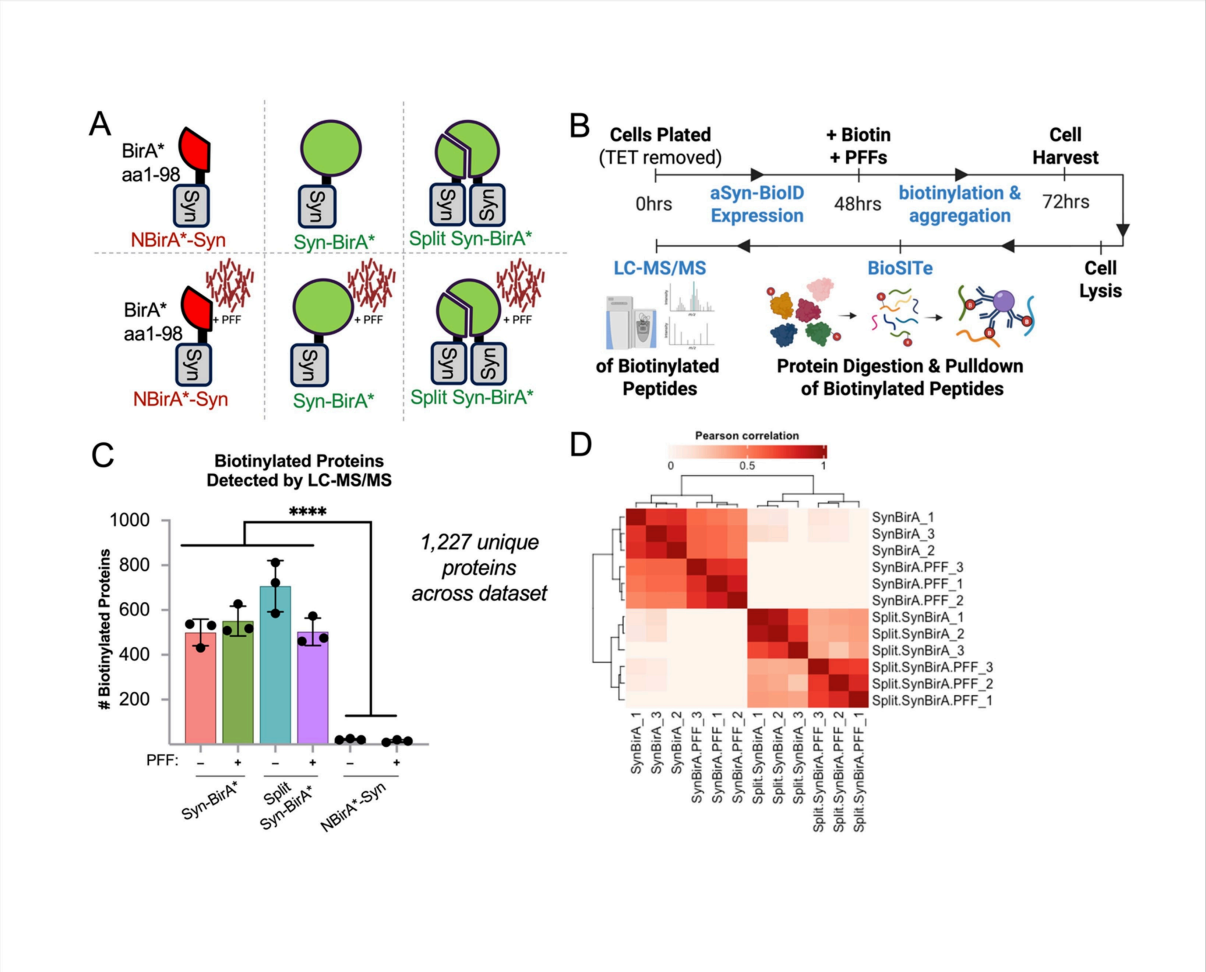
H4 Lysate Samples Analyzed by Proteomics **A.** Syn-BirA*, Split Syn-BirA* and NBirA*-Syn cells were cultured with PFFs (+ PFF) or with transfection reagent only (6 groups). Triplicates for each group (18 samples total) were harvested for mass spectrometry analysis. **B.** Timeline of experimental setup. **C.** Raw proteomics data reported 1,227 unique proteins, of which significantly more proteins were detected in Syn-BirA* and Split Syn-BirA* groups than NBirA*-Syn only groups. Significance was calculated via one-way ANOVA with post-hoc Tukey’s test; p-values < 0.0001 (****) are reported. **D.** Pearson correlation of processed data highlights similarity between replicates and construct type. Data processing removed proteins present in NBirA*-Syn groups or present in < 2 replicates in all groups, log transformed values, and imputed remaining missing values with a zero.

To generate cell lysates for mass spectrometry of proximal proteins, NBirA*-Syn, Syn-BirA* and Split Syn-BirA* cells were incubated with or without PFFs (Fig. 2A). After 48 hours of construct expression, excess biotin (50uM) and PFFs were added to cells *simultaneously* to enable biotinylation of proteins proximal to aSYN *during* the first 24 hours of aggregation. At the end of this incubation, cells were harvested and biotinylated proximal proteins in cell lysates were identified via BioSITe enrichment [34] and LC-MS/MS (Fig. 2B). In total we detected 1,277 unique proteins across all groups. As expected, significantly more proteins were detected in Syn-BirA* and Split Syn-BirA* groups than the negative control (Fig. 2C). All proteins detected in the negative control were removed during data processing. We performed a Pearson correlation analysis of post-processed data and demonstrate similarity between Syn-BirA* and Split Syn-BirA* replicates (Fig. 2D). In total, 581 proteins were identified as potential aSYN interactors.

**Figure 3.**
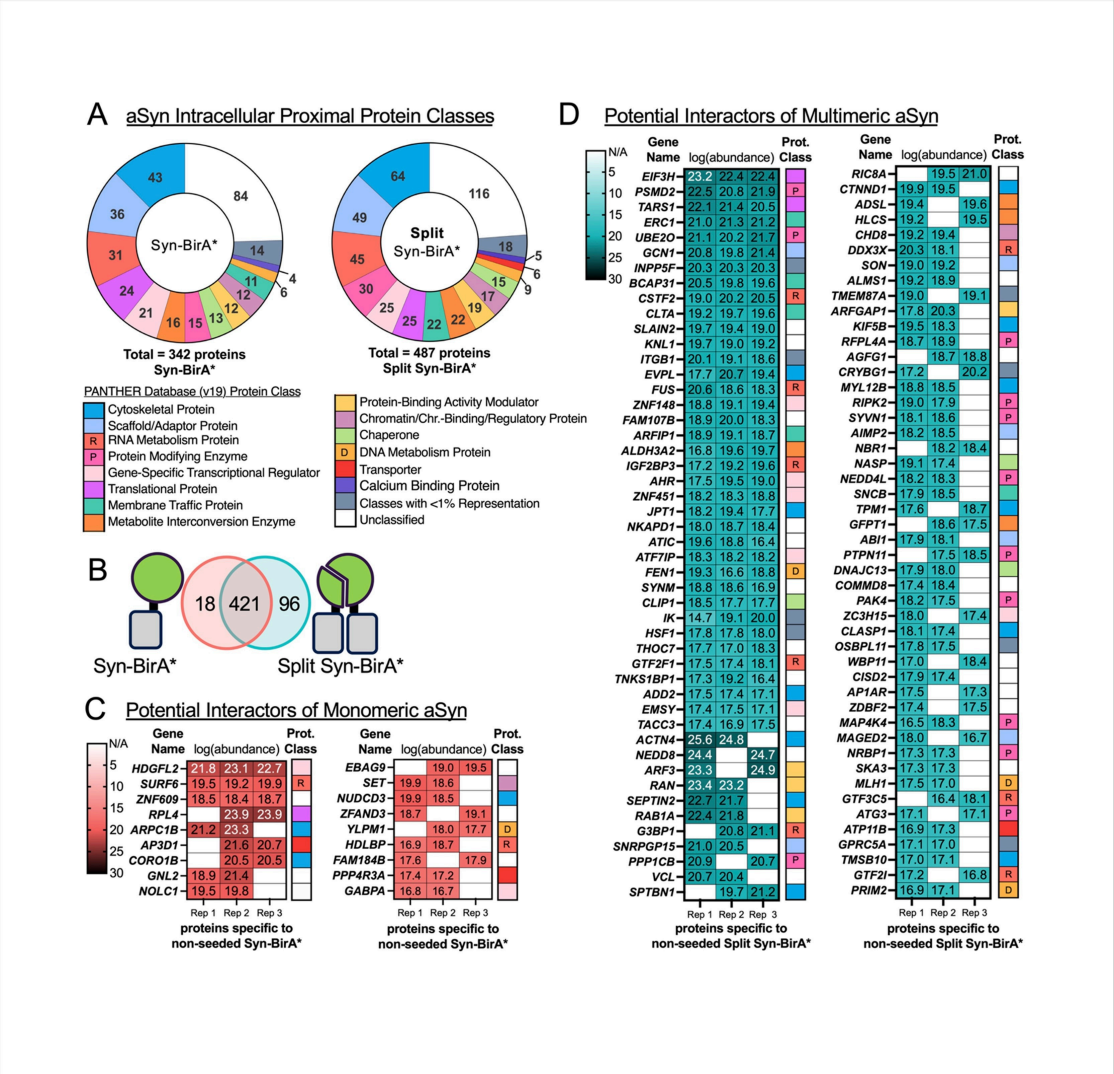
Potential Interactors of Unseeded aSYN **A.** Protein classes (identified by PANTHER Database) represented by unseeded Syn-BirA* potential interactors (left) and unseeded Split Syn-BirA* potential interactors (right). Proteins were included if they were present in ≥ 2 of 3 replicates. **B.** Proximal proteomes were compared between unseeded Syn-BirA* and unseeded Split Syn-BirA*. Proteins specific to one side of the comparison were present in ≥ 2 of 3 replicates of interest and absent in the opposing group. **C-D.** Gene names, transformed abundance values, and protein classes for 18 proteins specific to unseeded Syn-BirA* (C) and 96 proteins specific to unseeded Split Syn-BirA* (D) are listed.

At baseline, we detected 342 potential interactors of aSYN (Supplementary Fig. 2). Many of the identified proteins are associated with either cytoskeletal or scaffolding functions, RNA-regulation, regulation of gene expression, or enzymatic function (Fig. 3A, left). When we consider proteins that could be unique interactors of multimeric aSYN, we identified 487 proteins with specificity to multimeric aSYN (Supplementary Fig. 3). Of note, compared to the baseline profile of potential aSYN interactors (Fig. 3A, left), protein modifying enzymes represent a greater proportion of potential interactors of multimeric aSYN (represented by Split Syn-BirA*) (Fig. 3A, right). In fact, a comparison of the Syn-BirA* versus Split Syn-BirA* profiles (Fig. 3B) identifies 18 potential monomer-specific interactors (Fig. 3C) and 96 potential multimer-specific interactors (Fig. 3D). Many of the potentially multimer-specific interactors are protein modifying enzymes, such as: PSMD2, UBE2O, PPP1CB, RIPK2, SYVN1, NEDD4L, PTPN11, MAP4K4, NRBP1, and ATG3 (Fig. 3D).

**Figure 4.**
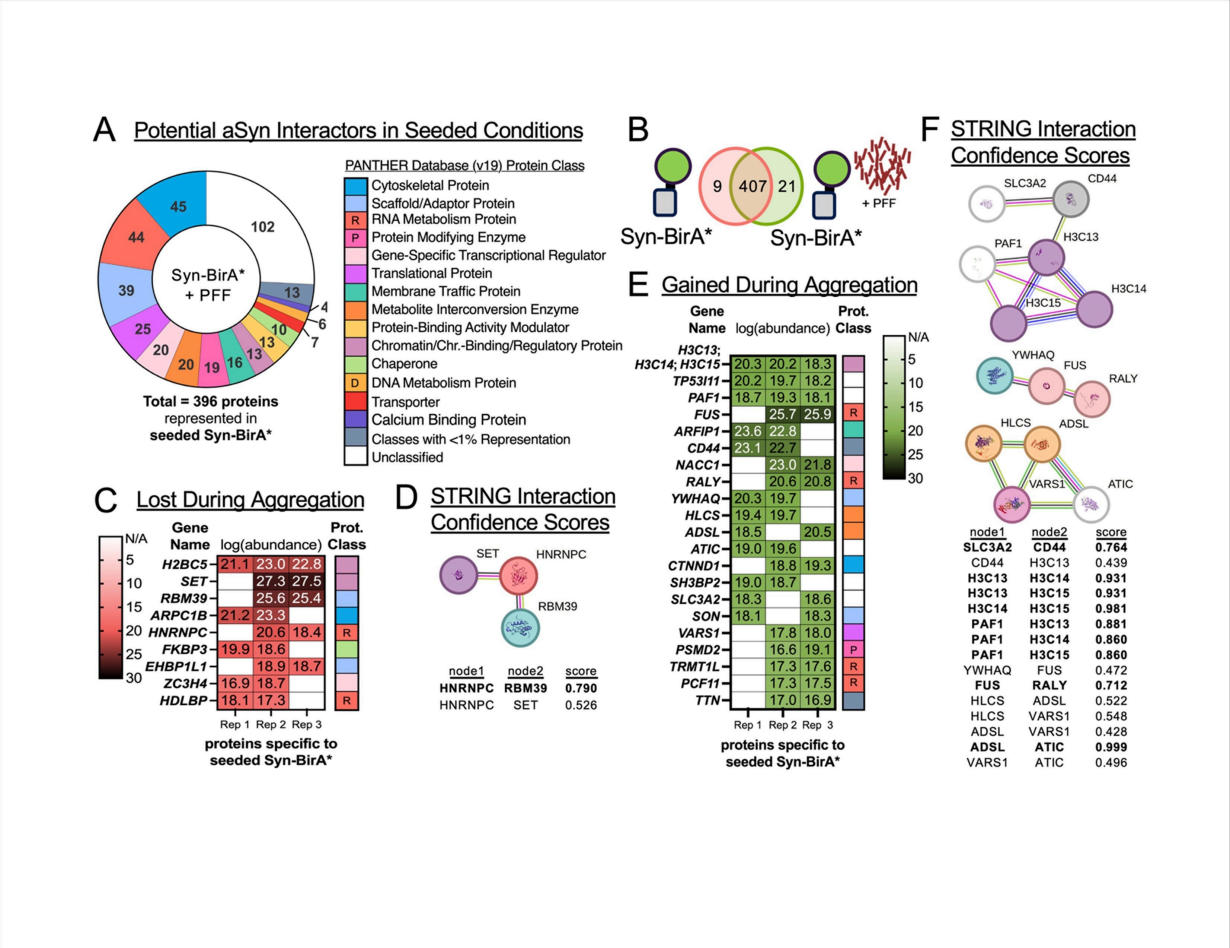
Potential Gained and Lost Interactors of aSYN During Aggregation **A.** Protein classes of all potential interactors of aggregating aSYN (seeded Syn-BirA*). Proteins included were identified in ≥ 2 of 3 replicates. **B.** Comparison of proximal proteomes from unseeded versus seeded Syn-BirA*. Proteins were specific to a group if they were identified in ≥ 2 of 3 replicates of the group of interest and absent in the opposing group. **C.** Gene names, transformed abundance values, and protein classes for 9 proteins specific to unseeded Syn-BirA*. **D.** STRING interaction confidence scores (medium confidence cutoff = 0.400) of potential interactions between the nine proteins specific to unseeded Syn-BirA*. STRING-designated high interaction confidence scores (0.700–0.899) and highest interaction confidence scores (> 0.900) are in bold. **E.** Gene names, transformed abundance values, and protein classes for 21 proteins specific to seeded Syn-BirA*. Genes *H3C13*, *H3C14*and *H3C15* encode one identified protein, Histone H3.2 (Accession ID: Q71DI3). **F.** STRING interaction confidence scores between the 21 proteins specific to seeded Syn-BirA*, using the same parameters described in (D).

Three hundred ninety-six (396) potential interactors of aggregating aSYN were identified, representing the same protein classes observed previously (Fig. 4A). Comparison of pathogenic and unseeded potential interactors (Fig. 4B) identified nine proteins specific to unseeded aSYN (Fig. 4C). It is worth novel consideration that the nine protein interactors specific to physiological aSYN represent interactions that are lost when aggregation occurs. Curiously, STRING interaction confidence scores suggest three of the nine proteins (SET, HNRNPC and RBM39) may interact with one another (Fig. 4D). Twenty-one (21) potential interactors were specific to pathogenic aSYN (Fig. 4E). The 21 proteins contain three groups of connected proteins and likely represent new interactions occurring with aSYN during the aggregation process. (Fig. 4F).

**Figure 5.**
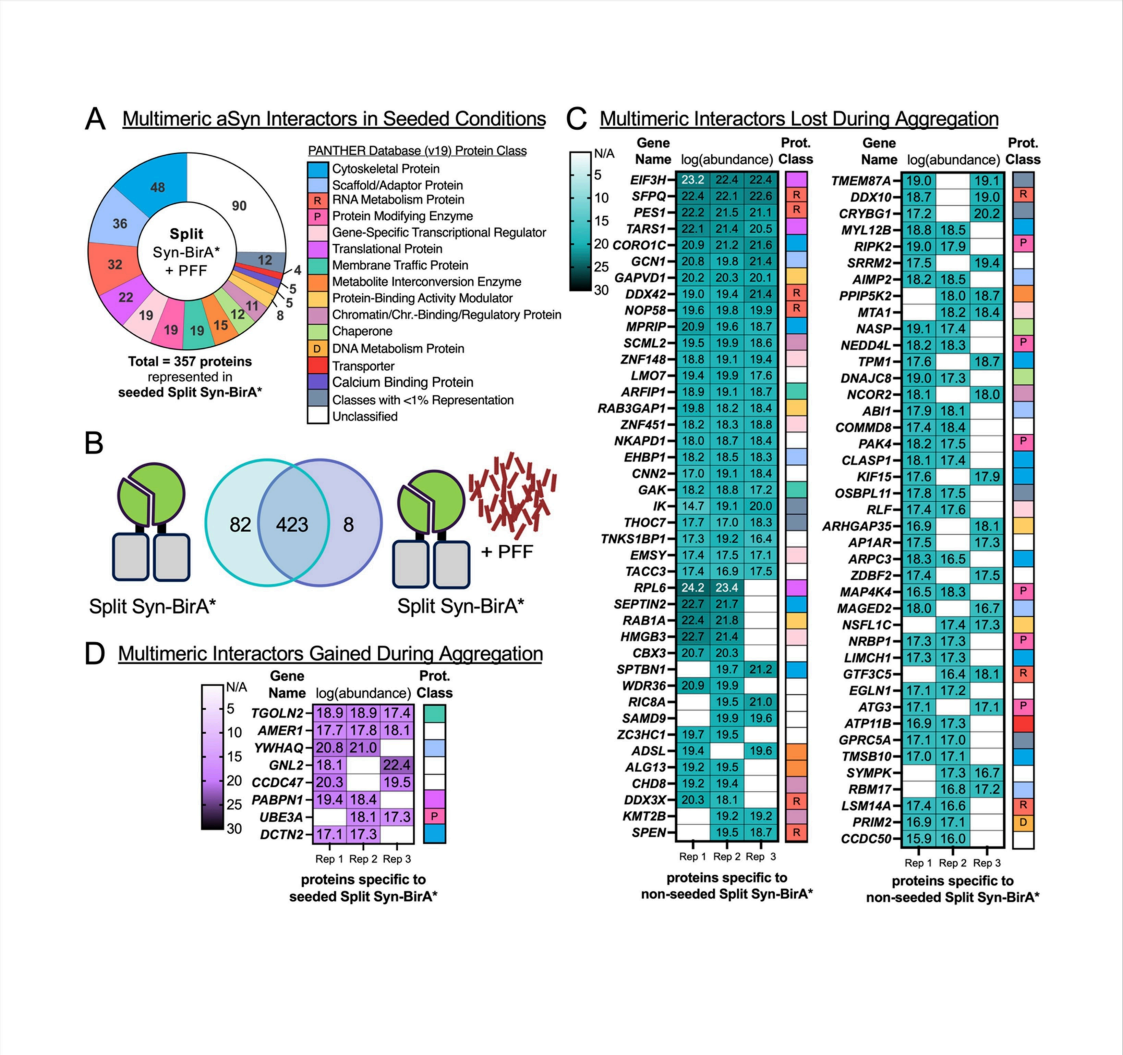
Changes to Multimeric aSYN Interactions in Response to Aggregation **A.** Protein classes of potential interactors specific to aggregating *multimeric* aSYN (seeded Split Syn-BirA*). Proteins included were identified in ≥ 2 of 3 replicates. **B.** Comparison of proximal proteomes from unseeded versus seeded Split Syn-BirA*. Proteins were specific to a group if they were identified in ≥ 2 of 3 replicates of the group of interest and absent in the opposing group. **C-D.** Gene names, transformed abundance values, and protein classes for 82 proteins specific to unseeded Split Syn-BirA* (C) and 8 proteins specific to seeded Split Syn-BirA* (D).

We next evaluated changes specific to multimers of aSYN during the pathogenic aggregation process. Of the 357 potential interactors of seeded, multimeric aSYN, we noted a decreased proportion of protein modifying enzymes and protein-binding activity modulators (Fig. 5A) compared to the unseeded state. Unseeded versus pathogenic Split Syn-BirA* data (Fig. 5B) reveals 82 proteins specific to unseeded aSYN multimers (Fig. 5C). Of these, the protein modifying enzymes RIPK2, PAK4, NEDD4L, MAP4K4, NRBP1 and ATG3 were all found in proximity to aSYN multimers, but not aSYN monomers (Fig. 3D). Protein-binding activity modulators (such as p47 encoded by *NSFL1C*) and RNA metabolism proteins (such as SFPQ) were also specific to unseeded states. All these proteins potentially lose interaction with aSYN multimers during aggregation. By contrast, we identified 8 proteins which represent a gained interaction with aggregating aSYN multimers (Fig. 5D). Half of these eight – TGOLN2, CCDC47, UBE3A and 14–3-3 theta isoform encoded by *YWHAQ* – are related to protein degradation and chaperone activity.

**Figure 6.**
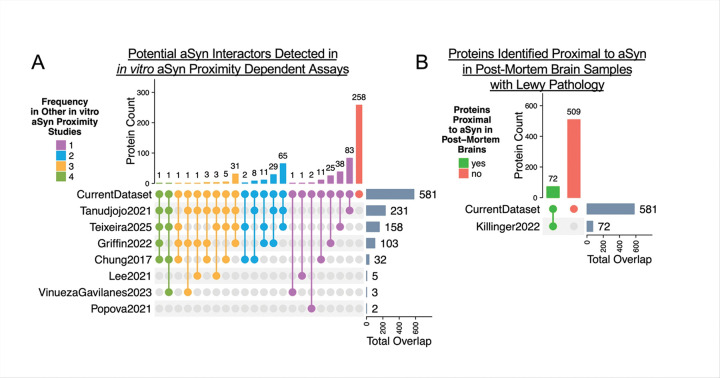
Comparison to Public Proximity-Dependent Datasets Bolsters Findings **A.** The current dataset was compared to publicly available *in vitro* proximity-labelling studies which differed in research scope, labelling method, and model system. Of the 581 proteins identified in the current dataset, 258 proteins were unique to this study (red), or found in one (161 proteins, purple), two (115 proteins, blue), three (45 proteins, yellow) or four (2 proteins, green) other studies. ‘Protein Count’ represents the number of proteins which were shared by the same datasets, portrayed by lines connecting filled circles. ‘Total Overlap’ represents the count of proteins detected both in this dataset and the public dataset of interest. **B.** Seventy-two (72) proteins identified in this study (green) were found proximal to aSYN in a study of post-mortem brain tissue with Lewy pathology.

As mentioned previously, proximity proteomics has been applied to query potential aSYN interactors in cell culture models and human tissue – albeit with varying research questions and experimental designs. To align our findings with previously generated data, we compared our dataset with publicly available datasets from aSYN proximity-labelling studies [24–29, 35]. Of the 581 proteins identified in the current study, 258 proteins (44.4%) were unique to this dataset, 161 proteins (27.7%) were reported in one other study, and 162 proteins (27.8%) were identified in multiple other studies (Fig. 6A, Supplementary Table). We also compared the current dataset to a study of proximal proteins of aSYN in post-mortem Lewy pathology [4]. Of 581 total proteins in the current dataset, 72 proteins (12%) were proximal to aSYN in Lewy bodies (Fig. 6B, Supplementary Table). These results provide confidence in the current data set, while also highlighting the variability associated with data obtained from different cell lines, different forms of aSYN (i.e. wildtype vs. mutant), and different methods of proximity tagging. Overall, the data obtained in this study contains a mix of known protein targets and novel candidates for future study.

## Discussion

Here we have established a split BirA* method to interrogate the proteome of aggregates associated with synucleinopathies. As of now, the mechanism(s) which trigger and exacerbate aSYN pathology are unclear, and the timing and purpose of Lewy body formation remains undefined. This study addresses these unknowns *in vitro* by identifying potential protein interactors of aSYN during the aggregation process. Here we identify 450 + potential protein interactors of unseeded aSYN and highlight specific changes in the interactome during aggregation. Importantly, for the first time, a split Syn-BirA* was used to discern protein interactors specific to multimeric aSYN. This study uncovers how the potential interactome of multimeric aSYN changes due to aggregation.

Analysis of the proximal proteome under physiological conditions shows that greater proportions of membrane trafficking proteins (vesicle coat proteins, SNARE proteins) and protein-modifying enzymes (proteases, ubiquitin-protein ligases, serine/threonine kinases) interact with multimeric aSYN (Fig. 3A). Increased proximity of membrane trafficking proteins – ERC1, BCAP31, CLTA, ARFIP1 (Fig. 3D) – to unseeded multimeric aSYN aligns with known association of physiological aSYN with cell membranes [36–38]. Similarly, the association of protein-modifying enzymes such as proteases and ubiquitin-protein ligases align with known intracellular quality control mechanisms [39] which degrade alpha-synuclein to maintain homeostasis.

Phosphorylation at serine 129 on aSYN is considered to be a marker of disease-associated aSYN, but recent studies suggest aSYN phosphorylation serves a physiological functional role in healthy cells [40]. Consistent with the latter concept, we detected serine/threonine kinases as likely interactors of unseeded aSYN multimers: PAK4, RIPK2, and MAP4K4. These kinases were not only found to be multimer-specific compared to monomeric aSYN (Fig. 3D), but they likely lost their interaction with aggregating multimeric aSYN (Fig. 5C). Thus, one could speculate that aberrant interactions of these kinases promote the conversion of physiological aSYN species into pathogenic forms.

In fact, PAK4 has been associated with Parkinson’s disease (PD): patient substantia nigra exhibited reduced PAK4 expression [41], *in vivo* neuronal ablation of PAK4 increased aSYN aggregation and PAK4 gain-of-function reduced aggregation [42], although this impact was suggested to be indirect. *MAP4K4* was recently suggested to be a risk gene [43] and a potential biomarker [44] for Parkinson’s disease. Our data suggest PAK4, RIPK2, and MAP4K4 may indeed interact with aSYN, but only multimeric forms. These interactions may be necessary to maintain healthy levels of phosphorylated aSYN multimers. Perhaps certain misfolded aSYN conformations prevent proper alignment of multimers with kinase active sites, exacerbating a feedback loop of poor interaction, and greater presence of pathogenic aSYN. Future studies must determine if these kinases actively function on aSYN multimers, and whether aberrant function of these kinases is sufficient to induce pathogenic aSYN aggregation.

Insulin resistance has been connected to onset of PD [45–47] and some positive clinical results are being reported from GLP-1 receptor agonist administration to Parkinson’s disease patients [48]. In the current study, IGF2R, a receptor for insulin-like growth factor, and IGF2BP3, a protein which binds to insulin like growth factor mRNA, were identified proximal to multimeric aSYN (Supplementary Fig. 3, and Fig. 3D). This supports the connection between PD and insulin signaling, and presents an intriguing potential link between unseeded multimeric aSYN and insulin-related proteins such as IGF2BP3.

We next evaluated global changes in potential interactors during aggregation. Three proteins – ARPC1B, HDLBP and SET – were potential interactors of monomeric aSYN (Fig. 3C) that lost proximity to aSYN upon the addition of PFFs (Fig. 4C). SET may interact with HNRNPC and RBM39 (Fig. 4D) as part of a mechanism specific to monomeric, physiological aSYN, which is lost during pathogenic aggregation. Future studies should investigate if aberrant interaction of proteins such as SET are associated with sporadic Lewy body disease.

A closer look at aggregating multimeric aSYN demonstrated gained potential interactors capable of clearing pathogenic proteins (Fig. 5D). TGOLN2 is involved in protein sorting and trafficking, potentially recycling aSYN oligomers back to endosomes or vesicle-based secretion for neuroprotection. CCDC47 is found in the endoplasmic reticulum and is linked to endoplasmic reticulum associated degradation (ERAD), UBE3A is an E3 ubiquitin ligase representative of another mechanism of protein degradation. 14–3-3 theta isoform (encoded by *YWHAQ*) is thought to demonstrate chaperone activity, addressing misfolded proteins. These data suggest gained interactions of multimeric aSYN serve functional roles in aggregate clearance. This bolsters the idea that *loss* of a homeostatic interaction may be more likely to set off aSYN aggregation than the gained interaction of a disruptive protein. This theory aligns with our conclusions regarding serine/threonine kinases.

One protein was consistently identified in cells treated with PFFs: 14–3-3, theta isoform (encoded by *YWHAQ*, Fig. 4E and Fig. 5D). The 14–3-3 protein family is ubiquitously expressed with a variety of functions, including chaperone activity [49]. Curiously, the theta isoform of 14–3-3 has demonstrated neuroprotective impacts in the substantia nigra of *in vivo* models challenged with aSYN PFFs [50]. Other 14–3-3 isoforms have been discovered as interactors of both wildtype and mutant aSYN [26]. Despite these observations, the contribution of the 14–3-3 protein family to neurodegeneration is considered ambiguous within the field.

Previous studies have suggested that aSYN alters RNA stability by sequestering RNA-related proteins in the cytoplasm [51]. Consistent with this, we uncovered notable global changes among RNA-related proteins during aggregation. Aggregating aSYN had increased proximity to RNA metabolism proteins (Fig. 4A): FUS, RALY, TRMT1L and PCF11 (Fig. 4E). FUS is an RNA-binding protein known to mislocalize to the cytoplasm in amyotrophic lateral sclerosis (ALS) and frontotemporal dementia (FTD). Interestingly, known interactors of FUS include 14–3-3 (encoded by *YWHAQ*) and RALY, both of which were identified as being proximal to aggregated aSYN (Fig. 4F) in our dataset. FUS was proximal to aSYN both in both pathogenic and unseeded multimer-specific groups. Perhaps multimeric aSYN – whether physiological or pathogenic – sequesters FUS in the cytoplasm, contributing to FUS pathology. Co-pathology of aSYN and FUS has not been commonly described with only one mention of insoluble FUS identified in dementia with Lewy body brain that we are aware of [52]. These data support the finding that multimeric aSYN and FUS may interact. The co-pathology of these two disease-associated proteins warrants new attention for follow-up.

BirA* enzymes label proteins in close proximity with a biotin moiety in the presence of excess biotin. However this does not mean that the proximal proteins actually interact with aSYN. Future experiments will be necessary to verify that proteins identified are true interactors of aSYN. Towards this end, we compared publicly available datasets from other proximity proteomics studies to our own dataset. First, public datasets were compiled from studies which similarly sought to identify potential interactors of aSYN *in vitro*, but in different cell types and with alternative output measures [24–29, 35]. Cell types spanned yeast, cortical rat neurons, hESC-derived neurons, and non-neuronal human cells. Wildtype aSYN, mutant aSYN or phosphorylated aSYN mimics were used. Some studies induced aggregation, while many did not. Additionally, different labelling techniques were used with protein labelling times as short as 1 minute and as long as 24 hours. Despite these differences, 55.5% of the proteins identified in the current study overlapped with proteins detected in published data, with the remaining 44.4% representing novel findings (Fig. 6A). Overall, the overlap provides confidence in our study and provides for the first time a comparison between of unseeded and pathogenic aSYN potential interactors. Next steps will be validating the interaction of proteins from this report with aSYN.

A limitation of this study is the use of aSYN overexpression in non-neuronal cells. Proteins involved in synaptic function may be underrepresented; and proteins proximal to aSYN in this model system may not be proximal in patient brains or may not be true interactors. To address this limitation, we compared the current dataset to a dataset of proteins proximal to aSYN in Lewy bodies. Twelve percent of the proteins identified in the current study were detected in proximity to aSYN in post-mortem Lewy pathology (Fig. 6B) [11]. While this comparison does not directly address the cell model limitation, it demonstrates that the current dataset includes relevant proteins that are destined to become a part of end-stage neuronal Lewy pathology.

Taken together, this study uncovers how aSYN interactions may change across conformation states, whether that be monomeric versus multimeric or physiological versus pathogenic. The observations suggest it is possible that a novel, important driver of spontaneous aSYN aggregation can be found by evaluating the impact of interactors of unseeded, multimeric aSYN. The data provide new insights on the connections between multimeric aSYN and kinases maintaining homeostasis, proteins involved in insulin resistance, and proteins involved in ALD/FTD such as FUS. Furthermore, the data highlight proteins which may contribute to spontaneous aSYN aggregation. Future studies will need to apply more physiologically relevant models, such as iPSC-derived neurons or brain organoids, to validate interactions proposed here, and to evaluate the functional significance of a protein to aSYN aggregation. These findings lay the groundwork for determining the role of Lewy pathology in Lewy body diseases and may also help biomarker development for prodromal stages of Lewy body diseases.

## Methods

### Plasmids and Constructs

BirA-R118G (BirA*; Addgene Plasmid #36047 [19]) was used to generate Syn-BirA*, Syn-CBirA* and NBirA*-Syn. Syn-BirA* with human alpha-synuclein inserted at NheI / BamHI restriction sites. Syn-CBirA* contains amino acids 99–321 fused to the carboxy terminal of aSYN, while NBirA*-Syn contains amino acids 1–98 of BirA and a myc tag fused to the N-terminus of aSYN. Constructs were subcloned into pTRE3G-BI-FRT [30], and used to generate stable cell lines.

### H4 Neuroglioma Cells Stably Expressing BirA* Constructs

Human H4 neuroglioma (ATTC, HTB-148) TetOFF cells compatible with the Flp-In system (Invitrogen, K6010–01 / −02) were previously described [30]. The following plasmids were used to create single-copy isogenic lines using the manufacturer’s protocol for integration and selection: pTRE3G-BI-FRT-Syn-BirA*, pTRE3G-BI-FRT-NBirA*-Syn, pTRE3G-BI-FRT-Syn-CBirA* or pTRE3G-BI-FRT-Split-Syn-BirA*.

### Cell Culture Maintenance

Cells were grown at 37 degrees Celsius with 5% CO_2_ in a humidified incubator. H4 TetOFF cells were grown in Opti-MEM + Glutamax media (Gibco, 51985–034) supplemented with final concentrations of 10% fetal bovine serum (Gibco, A52567–01), 200ug/mL geneticin (Gibco, 10131–035), and 300ug/mL zeocin (Invitrogen, R25001). Stably integrated H4 cell lines were grown in Opti-MEM + Glutamax media supplemented with final concentrations of 10% fetal bovine serum, 200ug/mL geneticin, and 200ug/mL hygromycin B (Invitrogen, 10687010). Tetracycline hydrocholoride (TET) (Sigma, T7660–5G) in water was added to media for a final concentration of 1ug/mL to suppress transgene expression when applicable.

### Pre-Formed Fibrils (PFF) Preparation and Addition

PFFs were generated in-house or purchased commercially (Stressmarq, SPR-322C; in Fig. 1D and Fig. 1E). In-house PFFs were generated using previously described protocols [31]: recombinant alpha-synuclein monomer (Proteos, RP-003) was diluted to 5mg/mL in PBS, then shaken for 7 days at 1000 RPM. In-house and commercial PFFs were aliquoted for one-time use to minimize freeze-thaws, following best practice guidelines [13].

At time of use, an aliquot of PFFs was diluted in sterile DPBS (Gibco, 14190–144) to reach 0.1ug/uL. PFFs were sonicated for 10 cycles of 30 seconds ON / 30 seconds in a Biorupter Plus or Pico. Sonicated PFFs were prepared for transfection via lipofectamine 2000 (Invitrogen, 11668019) in Opti-MEM (Gibco, 11058–021) following manufacturer protocols.

### Biotin Preparation and Addition

Biotin (Sigma, B4639–500mg) was added to cell media at a final concentration of 50uM.

### Streptavidin Pulldown of Biotinylated Proteins

For each group, cells were seeded in 6-well plates (Corning, 3516) in TET-free media. At 24 hours, biotin-media was prepared and added to cells. Cells were transfected with PFFs using lipofectamine 2000. After 24 hours, cells were rinsed 2x with cold sterile DPBS, scraped and pelleted (Centrifugation at 1000g for 5 min, at 4 degrees Celsius) and stored at −80 degrees Celsius. At time of lysis, cell pellets were resuspended in 400ul urea lysis buffer [8M urea (Sigma, U0631–1KG), 50mM Tris-HCl pH 7.5 (Invitrogen, 15567–027), EDTA-free protease inhibitor (Roche, 11836170001), 1mM DTT (Sigma, 43816–10mL) in H20], and sonicated for 10 cycles of 30 seconds ON / 30 seconds OFF. Samples were centrifuged at 16k × g for 10 minutes at 4 degrees Celsius and supernatant (cell lysate) was extracted.

All pulldown centrifugation steps were at 1000g for 2 minutes. All rotations were for 8 minutes, unless otherwise specified. Streptavidin beads (Cytvia, 17511301) were rotated in urea lysis buffer. Beads were pelleted and resuspended in cell lysate (“input”) and rotated overnight at 4 degrees Celsius. Beads were pelleted; supernatant (“flow-through”) was stored. Beads went through multiple rounds of centrifugation, resuspension and rotation in the following solutions: thrice in wash buffer [8M urea, 50mM Tris-HCl pH 7.5 in H20], twice in 50mM Tris-HCl pH 7.5 in H20, and thrice in 50mM ammonium bicarbonate in H20. Beads were finally resuspended in elution buffer [20mM biotin (Sigma Aldrich, B4639–500mg), 5% SDS (Sigma, L3771–500G), 50mM HEPES (Gibco, 15630–106)] and heated to 95 degrees Celsius for 10 mins. Beads were pelleted and supernatant (“eluate”) was stored.

### Cell Lysis for Western Blot

Cell pellets were resuspended in non-urea containing lysis buffer [150mM NaCl (Sigma, S7653–1KG), 50mM Tris-Cl pH 7.4, 0.1% Triton-X (Sigma, X100–500mL), Protease Inhibitor (Roche, 11836153001)], and sonicated for 10 cycles of 30 seconds ON / 30 seconds OFF. Samples were centrifuged (16k × g, 10 minutes, 4 degrees Celsius) and supernatant (cell lysate) was stored.

### Western Blot

Unless otherwise stated, 10ug of cell lysate, 2.5ug of pulldown input, 2.5ug of pulldown flow-through or 0.5ug of pulldown eluate were loaded per well with protein concentrations estimated using bicinchoninic acid assay (Thermo, 23225). Proteins were separated on stain free gels (BioRad, 4568124 or 4568126) in ice cold running buffer (BioRad, 1610772), running at 300V for 23 minutes. Dual Color (BioRad, 1610374) or Precision Plus WesternC (BioRad, 1610376) ladders were used. Proteins were transferred to 0.22um nitrocellulose membranes using BioRad’s TransBlot Turbo Transfer System and ice-cold transfer buffer (BioRad, 10026938).

Membranes were blocked in 3% BSA (Sigma, A7906–50G) in TBS with 0.2% Tween (Sigma, P2287–500mL) and 0.1% SDS (Sigma, L3771–500G) for 1 hour at room temperature. Anti-Syn membranes were probed with MJFR1 (1:10K) for 1 hour at room temperature or overnight at 4 degrees Celsius, prior to being washed (3 × 5 min in TBS-T). Membranes were incubated with anti-rabbit-HRP (Southern Biotech, 4010–05) for anti-aSYN blots or anti-streptavidin-HRP (Southern Biotech, 7100–05) for anti-biotin blots for 1 hour at room temperature. Membranes were washed (3 × 5 min in TBS-T). HRP substrate (Millipore, WBKLS0500) was applied to membranes for 20–30 seconds immediately prior to imaging on BioRad’s ChemiDoc MP Imaging System. Image analysis and quantification was performed in Image Lab 6.1.

### Cell Culture for Immunocytochemistry

Cells were seeded in 24-well plates (Cellvis, P24–1.5P) in TET-free media. At 24 hours (Fig. 1E) or 48 hours (Fig. 1C), biotin-media was prepared and added to cells via media change. In Fig. 1E, PFF transfection mixture was added to cells immediately after media change. After another 24 hours, cells were washed with cold sterile DPBS, fixed with 4% PFA (Sigma-Aldrich, 441244–1KG dissolved in PBS) for 15 minutes, and washed again with DPBS.

### Immunocytochemistry

Cells were permeabilized in 0.1% PBS-Tx for 5 minutes (Fig. 1C) or 0.5% PBS-Tx for 20 minutes (Fig. 1E), then blocked for 1 hour in 5% BSA in 0.1% PBS-Tx (Fig. 1C) or 1.5% normal goat serum (NGS; Sigma, G9023) in PBS (Fig. 1E). Cells incubated in primary antibody for 1 hour at room temperature in anti-aSYN primary antibody (1:2000; 4B12; Biolegend, 807802) only for Fig. 1C, or both anti-aSYN primary antibody and anti-pSyn primary antibody (1:500; D1R1R, Cell Signaling, 23706S) for Fig. 1E. Cells were washed (3 × 5 minutes in PBS). Cells were then incubated for 1 hour at room temperature in secondary antibodies (1:500; anti-mouse 488; Invitrogen, A11001) and neutravidin (1:500; NeutrAvidin Protein Dylight 650; Invitrogen, #84607) for Fig. 1C, or secondary antibodies only (1:500; anti-mouse 488; Invitrogen, A11001 and 1:500; anti-rabbit 555; Invitrogen, A32732) for Fig. 1E. 1x Hoechst 33342 (Invitrogen, H3570) in PBS was applied to cells for up to 5 minutes, then cells were washed and stored in fresh PBS. Figure 1C images were acquired with Zeiss AxioObserver at 4x magnification. Figure 1E Z-stack images were taken on the Zeiss LSMP900 confocal at 20x magnification. Images are grouped to maintain consistent exposure and display settings. Z-stacks were merged using Extended Depth of Focus (method: wavelets; z-stack alignment: normal) in Zen Blue software (Zeiss).

### Cell Culture for BioSITe and LC-MS/MS

Triplicates were prepared for each group analyzed by proteomics. For each replicate, cells were plated in three 15cm dishes (CytoOne, CC7682–3614), in TET-free media. After 48 hours, biotin-media was added to cells via media change. PFF transfection mixture was then immediately added to cells. After 24 hours, cells were rinsed 2x with cold sterile DPBS, scraped and pelleted (Centrifugation at 1000g for 5 min, at 4 degrees Celsius). Cell pellets were stored at −80 degrees Celsius. A fraction of the cell mixture was saved for internal experiments. Remaining pellet was sent to the Mayo Clinic Rochester Proteomics Core for BioSITe and LC-MS/MS analysis.

### BioSITe: Sample Preparation

Cell pellets were suspended in 2.5mls 0.1% SDSD/ 10mM TEAB pH 8.5 with Benzonase, 1mM MgCl2, and HALT protease/phosphatase inhibitors and lysed using a BioRuptor sonication system followed by protein concentration determination using the Pierce BCA assay. Volumes corresponding to 4 mg from each sample were lyophilized then solubilized in 450μl 5% SDS / 50mM triethylammonium bicarbonate (TEAB) pH 8.5 buffer and processed using an S-trap Midi device (Protifi.com) following the vendor recommended protocol reducing with 10mM TCEP with heat at 95°C for 10 minutes, cooling to room temperature and alkylating with 10mM iodoacetamide for 20minutes in the dark, followed with the addition of phosphoric acid to create the suspension and addition to the device for trapping and washing. A volume of 350ul 50mM TEAP pH 8.5 with 1:20 wt:wt ratio of Worthington trypsin is added to the device and incubated at 37°C overnight. The peptides are eluted with serial additions and spins of 500ul 50mM TEAB, 500μl 0.2% formic acid and 500ul 50% acetonitrile / 0.1% trifluoroacetic acid (TFA) into a single collection tube and lyophilized.

### BioSITe: Biotin Peptide Enrichment

For biotin peptide enrichment, the lyophilized peptides are solubilized in 400μl 50mM Tris, pH 7.4 / 150mM NaCl, 0.5% Triton X-100 and incubated with anti-biotin-agarose beads (Bethyl Laboratories) for 90minutes at 4°C in a Pierce spin column. The beads were washed with 3 × 600ul 50mM Tris washes followed with 4 × 600μl water washes and the captured biotin peptides collected with 2 × 200μl 0.2% TFA elution steps followed with 2 × 100μl 0.2%TFA / 40% acetonitrile steps which are pooled and lyophilized.

### LC-MS/MS: Mass Spectrometry Data Acquisition

The lyophilized biotin enriched peptides were analyzed by nanoLC-tandem mass spectrometry using a Thermo Scientific Exploris 480 Orbitrap mass spectrometer (Thermo Scientific, Bremen, Germany) coupled to a Vanguish Neo UHPLC system with the A solvent as 0.1% formic acid in 98% water / 2% acetonitrile and the B solvent as 0.1% formic acid in 80% acetonitrile / 10% isopropanol / 10% water. The peptides were solubilized in 0.2% formic acid / 0.1% TFA and pumped onto a PepMap Neo C18, 5μm, 300μm × 5mm trap with 0.1% formic acid / 0.05% TFA at a flow rate of 8ml / minute. The trap was placed in line with a 75um × 25cm C18, 1.7μm IonOpticks Ultimate TS nano column heated to 50°C and the peptides separated at a flow rate of 300nl/minute with a gradient of 2%B to 30%B over 120minutes followed with a 10-minute ramp to 90%B and hold for 10 minutes, then equilibration at 2%B. The Exploris 480 was set for data dependent acquisition with a 2 second cycle time. The MS1 survey scan range was 360–1500 m/z at resolution 120,000 (@ 200m/z) with the automatic gain control (AGC) set to allow up to 2×10^6^ ions (200%) and the maximum ion inject time set to auto. Precursor ions in the scan range of 350–1500 m/z, with positive charge states from 2–4, were sequentially selected with an isolation window of 1.2 m/z and fragmented by high energy collisional dissociation (HCD) with a NCE setting of 30%. The MS/MS scans were acquired at resolution 15,000 with the AGC setting at 80% (8×10^4^ ions) and the max ion inject time set to 50ms. The dynamic exclusion feature was used to prevent any ion within a 20ppm window of the selected ion, from being chosen again for MS/MS fragmentation for at least 30 seconds.

### LC-MS/MS: Database Searching and Analysis

Data were searched using Swissprot Human Reviewed protein sequences (20,347 entries, version 2023_01) in Proteome Discoverer 3.0 (Thermo Scientific). Enzyme specificity was set to full trypsin with 3 missed cleavages and precursor and fragment was tolerance was set to 10ppm and 0.02 Da respectively. Carbamidomethylation of cysteine was set as a static modification and oxidation of methionine and biotin at lysine as variable modifications. The peptide identification results were filtered at 1% FDR and the biotinylated peptide abundance values were calculated with the precursor ion intensities.

### Proteomic Data Analysis

See supplementary information for raw data, excluded proteins and processed data. Abundances for 6 biological triplicates were analyzed with R (version 4.4.2). Raw abundances were compiled (Supplementary Table ‘Raw Protein Data’ excel sheet). Proteins with non-zero abundances in any negative control sample were removed (Supplementary Table ‘Excluded NBirA Proteins’ sheet). The remaining data (Supplementary Table ‘Input for DEP Processing’ sheet) were used for analysis using the DEP Package (version 1.26.0) [32], designed for label free quantitation analysis. DEP processing filtered out proteins present in < 2 replicates in all groups (Supplementary Table ‘Excluded 0 or 1 Rep’ sheet). The remaining proteins (Supplementary Table ‘Post Exclusion, Remain Proteins’ sheet) had abundance levels normalized and imputed (missing value = 0). Filtered and normalized data are exported in the ‘Post DEP Scaled Final Dataset’ excel sheet and are reported in the main figures.

In pie charts, proteins present in ≥ 2 of 3 replicates were considered a potential interactor. In Venn diagrams, proteins present in ≥ 2 of 3 replicates and *not appearing at all* in the alternate group were considered a specific interactor. Proteins present in ≥ 2 of 3 replicates, and *1 + replicate* of the alternate group were allocated into the shared category.

### Protein Classes via PANTHER Version 19

Accession IDs were input into the PANTHER Classification System Version 19.0 (https://pantherdb.org), accessed on June 1, 2025. Parameters were set to: Select List Type: ID List; Select Organism: Homo Sapiens; Select Analysis: Functional Classification Viewed in Gene List. For each PANTHER Protein Class displayed, the largest parent protein class was noted and reported. ‘Unclassified’ refers to Accession IDs with no protein class reported. Two accession IDs were not found in the database: Q9Y2D5 with gene name *PALM2AKAP2* and P42167 with gene name *TMPO.* Only one gene name appeared under more than one accession ID: *TMPO*. One accession ID linked to *TMPO* had a PANTHER protein classification; the other was not found.

### STRING Protein Interaction Confidence Scores

Interaction confidence scores were queried within the STRING database (https://string-db.org) using Accession IDs on August 4, 2025. Conversion to gene names was automatically done by the STRING platform. Minimum interaction score was set to medium confidence (0.400). Organism species was *Homo sapiens*.

### Comparison of Datasets via Upset Plot

Data were collected from published reports. Accession IDs were converted to gene names when necessary. Combined data were analyzed in R via ComplexHeatmap (version 2.20.0), UpSetR (version 1.4.0) and readxl packages (version 1.4.3). See ‘Supplementary Tables’ files for compilation of detected proteins from datasets.

### Statistical Analysis:

Statistics were calculated by Graphpad Prism (version 10.4.1). Data in Fig. 2C passed the Shapiro-Wilk Normality Test and were analyzed with ordinary one-way ANOVA with post-hoc Tukey’s test. p-values < 0.0001 (****) are reported.

## Supplementary Material

Supplementary Files

This is a list of supplementary files associated with this preprint. Click to download.

• SupplementaryTables.xlsx

• SupplementaryFigures.pdf

## Data Availability

The datasets generated during and/or analyzed during the current study are available in the supplementary information provided.

## References

[1] Polymeropoulos M.H., Lavedan C., Leroy E., Ide S.E., Dehejia A., Dutra A., Pike B., Root H., Rubenstein J., Boyer R., Stenroos E.S., Chandrasekharappa S., Athanassiadou A., Papapetropoulos T., Johnson W.G., Lazzarini A.M., Duvoisin R.C., Di Iorio G., Golbe L.I., and Nussbaum R.L.. (1997). Mutation in the α-Synuclein Gene Identified in Families with Parkinson’s Disease. science, 276(5321), 2045–2047. 9197268 10.1126/science.276.5321.2045

[2] Polymeropoulos M.H., Higgins J.J., Golbe L.I., Johnson W.G., Ide S.E., Di Iorio G., Sanges G., Stenroos E.S., Pho L.T., Schaffer A.A., Lazzarini A.M., Nussbaum R.L., and Duvoisin R.C.. (1996). Mapping of a Gene for Parkinson’s Disease to Chromosome 4q21-Q23. Science, 274(5290), 1197–9. 10.1126/science.274.5290.1197 8895469

[3] Spillantini M.G., Crowther R.A., Jakes R., Hasegawa M., and Goedert M.. (1998). α-Synuclein in Filamentous Inclusions of Lewy Bodies from Parkinson’s Disease and Dementia with Lewy Bodies. Proceedings of the National Academy of Sciences, 95(11), 6469–6473. 10.1073/pnas.95.11.6469 PMC27806 9600990

[4] Spina S., La Joie R., Petersen C., Nolan A.L., Cuevas D., Cosme C., Hepker M., Hwang J.-H., Miller Z.A., Huang E.J., Karydas A.M., Grant H., Boxer A.L., Gorno-Tempini M.L., Rosen H.J., Kramer J.H., Miller B.L., Seeley W.W., Rabinovici G.D., and Grinberg L.T.. (2021). Comorbid Neuropathological Diagnoses in Early Versus Late-Onset Alzheimer’s Disease. Brain, 144(7), 2186–2198. 10.1093/brain/awab099 33693619 PMC8502474

[5] Klos K., Ahlskog J., Josephs K., Apaydin H., Parisi J., Boeve B., DeLucia M., and Dickson D.. (2006). α-Synuclein Pathology in the Spinal Cords of Neurologically Asymptomatic Aged Individuals. Neurology, 66(7), 1100–1102. 16606927 10.1212/01.wnl.0000204179.88955.fa

[6] Gibb W.R. and Lees A.J.. (1988). The Relevance of the Lewy Body to the Pathogenesis of Idiopathic Parkinson’s Disease. Journal of Neurology, Neurosurgery & Psychiatry, 51(6), 745–752. 10.1136/jnnp.51.6.745 2841426 PMC1033142

[7] Gibb W.R. (1986). Idiopathic Parkinson’s Disease and the Lewy Body Disorders. Neuropathol Appl Neurobiol, 12(3), 223–234. 10.1111/j.1365-2990.1986.tb00136.x 3016582

[8] DelleDonne A., Klos K.J., Fujishiro H., Ahmed Z., Parisi J.E., Josephs K.A., Frigerio R., Burnett M., Wszolek Z.K., Uitti R.J., Ahlskog J.E., and Dickson D.W.. (2008). Incidental Lewy Body Disease and Preclinical Parkinson Disease. Arch Neurol, 65(8), 1074–1080. 10.1001/archneur.65.8.1074 18695057

[9] Bloch A., Probst A., Bissig H., Adams H., and Tolnay M.. (2006). Alpha-Synuclein Pathology of the Spinal and Peripheral Autonomic Nervous System in Neurologically Unimpaired Elderly Subjects. Neuropathol Appl Neurobiol, 32(3), 284–95. 10.1111/j.1365-2990.2006.00727.x 16640647

[10] Frigerio R., Fujishiro H., Ahn T.B., Josephs K.A., Maraganore D.M., DelleDonne A., Parisi J.E., Klos K.J., Boeve B.F., Dickson D.W., and Ahlskog J.E.. (2011). Incidental Lewy Body Disease: Do Some Cases Represent a Preclinical Stage of Dementia with Lewy Bodies? Neurobiol Aging, 32(5), 857–63. 10.1016/j.neurobiolaging.2009.05.019 19560232 PMC3366193

[11] Killinger B.A., Marshall L.L., Chatterjee D., Chu Y., Bras J., Guerreiro R., and Kordower J.H.. (2022). In Situ Proximity Labeling Identifies Lewy Pathology Molecular Interactions in the Human Brain. Proceedings of the National Academy of Sciences, 119(5), e2114405119. 10.1073/pnas.2114405119 PMC8812572 35082147

[12] Mazzetti S., De Leonardis M., Gagliardi G., Calogero A.M., Basellini M.J., Madaschi L., Costa I., Cacciatore F., Spinello S., Bramerio M., Cilia R., Rolando C., Giaccone G., Pezzoli G., and Cappelletti G.. (2020). Phospho-Hdac6 Gathers into Protein Aggregates in Parkinson’s Disease and Atypical Parkinsonisms. Front Neurosci, 14, 624. 10.3389/fnins.2020.00624 32655357 PMC7324673

[13] Leverenz J.B., Umar I., Wang Q., Montine T.J., McMillan P.J., Tsuang D.W., Jin J., Pan C., Shin J., Zhu D., and Zhang J.. (2007). Proteomic Identification of Novel Proteins in Cortical Lewy Bodies. Brain Pathology, 17(2), 139–45. 10.1111/j.1750-3639.2007.00048.x 17388944 PMC8095629

[14] Xia Q., Liao L., Cheng D., Duong D.M., Gearing M., Lah J.J., Levey A.I., and Peng J.. (2008). Proteomic Identification of Novel Proteins Associated with Lewy Bodies. Front Biosci, 13, 3850–6. 10.2741/2973 18508479 PMC2663966

[15] Martinez-Valbuena I., Swinkin E., Santamaria E., Fernandez-Irigoyen J., Sackmann V., Kim A., Li J., Gonzalez-Latapi P., Kuhlman G., Bhowmick S.S., Visanji N.P., Lang A.E., and Kovacs G.G.. (2022). α-Synuclein Molecular Behavior and Nigral Proteomic Profiling Distinguish Subtypes of Lewy Body Disorders. Acta Neuropathologica, 144(2), 167–185. 10.1007/s00401-022-02453-0 35748929

[16] Shahmoradian S.H., Lewis A.J., Genoud C., Hench J., Moors T.E., Navarro P.P., Castaño-Díez D., Schweighauser G., Graff-Meyer A., Goldie K.N., Sütterlin R., Huisman E., Ingrassia A., Gier Y., Rozemuller A.J.M., Wang J., Paepe A., Erny J., Staempfli A., Hoernschemeyer J., Großerüschkamp F., Niedieker D., El-Mashtoly S.F., Quadri M., Van I.W.F.J., Bonifati V., Gerwert K., Bohrmann B., Frank S., Britschgi M., Stahlberg H., Van de Berg W.D.J., and Lauer M.E.. (2019). Lewy Pathology in Parkinson’s Disease Consists of Crowded Organelles and Lipid Membranes. Nat Neurosci, 22(7), 1099–1109. 10.1038/s41593-019-0423-2 31235907

[17] Kalia L.V. and Lang A.E.. (2015). Parkinson’s Disease. Lancet, 386(9996), 896–912. 10.1016/s0140-6736(14)61393-3 25904081

[18] Petyuk V.A., Yu L., Olson H.M., Yu F., Clair G., Qian W.-J., Shulman J.M., and Bennett D.A.. (2021). Proteomic Profiling of the Substantia Nigra to Identify Determinants of Lewy Body Pathology and Dopaminergic Neuronal Loss. J Proteome Res, 20(5), 2266–2282. 10.1021/acs.jproteome.0c00747 33900085 PMC9190253

[19] Roux K.J., Kim D.I., Raida M., and Burke B.. (2012). A Promiscuous Biotin Ligase Fusion Protein Identifies Proximal and Interacting Proteins in Mammalian Cells. J Cell Biol, 196(6), 801–10. 10.1083/jcb.201112098 22412018 PMC3308701

[20] Kim D.I., Jensen S.C., Noble K.A., Kc B., Roux K.H., Motamedchaboki K., and Roux K.J.. (2016). An Improved Smaller Biotin Ligase for Bioid Proximity Labeling. Mol Biol Cell, 27(8), 1188–1196. 10.1091/mbc.E15-12-0844 26912792 PMC4831873

[21] Branon T.C., Bosch J.A., Sanchez A.D., Udeshi N.D., Svinkina T., Carr S.A., Feldman J.L., Perrimon N., and Ting A.Y.. (2018). Efficient Proximity Labeling in Living Cells and Organisms with Turboid. Nat Biotechnol, 36(9), 880–887. 10.1038/nbt.4201 30125270 PMC6126969

[22] Rhee H.-W., Zou P., Udeshi N.D., Martell J.D., Mootha V.K., Carr S.A., and Ting A.Y.. (2013). Proteomic Mapping of Mitochondria in Living Cells Via Spatially Restricted Enzymatic Tagging. Science, 339(6125), 1328–1331. 10.1126/science.1230593 23371551 PMC3916822

[23] Lam S.S., Martell J.D., Kamer K.J., Deerinck T.J., Ellisman M.H., Mootha V.K., and Ting A.Y.. (2015). Directed Evolution of Apex2 for Electron Microscopy and Proximity Labeling. Nat Methods, 12(1), 51–54. 10.1038/nmeth.3179 25419960 PMC4296904

[24] Popova B., Galka D., Häffner N., Wang D., Schmitt K., Valerius O., Knop M., and Braus G.H.. (2021). α-Synuclein Decreases the Abundance of Proteasome Subunits and Alters Ubiquitin Conjugates in Yeast. Cells, 10(9), 2229. 10.3390/cells10092229 34571878 PMC8468666

[25] Lee J.-Y., Kim H., Jo A., Khang R., Park C.-H., Park S.-J., Kwag E., and Shin J.-H.. (2021). α-Synuclein A53t Binds to Transcriptional Adapter 2-Alpha and Blocks Histone H3 Acetylation. Int J Mol Sci, 22(10). 10.3390/ijms22105392 PMC8161267 34065515

[26] Vinueza-Gavilanes R., Bravo-González J.J., Basurco L., Boncristiani C., Fernández-Irigoyen J., Santamaría E., Marcilla I., Pérez-Mediavilla A., Luquin M.R., Vales A., González-Aseguinolaza G., Aymerich M.S., Aragón T., and Arrasate M.. (2023). Stabilization of 14–3-3 Protein-Protein Interactions with Fusicoccin-a Decreases Alpha-Synuclein Dependent Cell-Autonomous Death in Neuronal and Mouse Models. Neurobiology of Disease, 183, 106166. 10.1016/j.nbd.2023.106166 37245833

[27] Griffin T.A., Schnier P.D., Cleveland E.M., Newberry R.W., Becker J., and Carlson G.A.. (2023). Fibril Treatment Changes Protein Interactions of Tau and α-Synuclein in Human Neurons. Journal of Biological Chemistry, 299(3), 102888. 10.1016/j.jbc.2023.102888 36634849 PMC9978635

[28] Tanudjojo B., Shaikh S.S., Fenyi A., Bousset L., Agarwal D., Marsh J., Zois C., Heman-Ackah S., Fischer R., Sims D., Melki R., and Tofaris G.K.. (2021). Phenotypic Manifestation of α-Synuclein Strains Derived from Parkinson’s Disease and Multiple System Atrophy in Human Dopaminergic Neurons. Nature Communications, 12(1), 3817. 10.1038/s41467-021-23682-z PMC8217249 34155194

[29] Teixeira M., Sheta R., Musiol D., Ranjakasoa V., Loehr J., Lambert J.-P., and Oueslati A.. (2025). Combining Light-Induced Aggregation and Biotin Proximity Labeling Strongly Implicates Endolysosomal Proteins in Early α-Synuclein Oligomerization. iScience. 10.1016/j.isci.2025.112823 PMC12221657 40612512

[30] Moussaud S., Malany S., Mehta A., Vasile S., Smith L.H., and McLean P.J.. (2015). Targeting α-Synuclein Oligomers by Protein-Fragment Complementation for Drug Discovery in Synucleinopathies. Expert Opin Ther Targets, 19(5), 589–603. 10.1517/14728222.2015.1009448 25785645 PMC4608017

[31] Polinski N.K., Volpicelli-Daley L.A., Sortwell C.E., Luk K.C., Cremades N., Gottler L.M., Froula J., Duffy M.F., Lee V.M.Y., Martinez T.N., and Dave K.D.. (2018). Best Practices for Generating and Using Alpha-Synuclein Pre-Formed Fibrils to Model Parkinson’s Disease in Rodents. J Parkinsons Dis, 8(2), 303–322. 10.3233/jpd-171248 29400668 PMC6004926

[32] Zhang X., Smits A.H., Van Tilburg G.B., Ovaa H., Huber W., and Vermeulen M.. (2018). Proteome-Wide Identification of Ubiquitin Interactions Using Ubia-Ms. Nat Protoc, 13(3), 530–550. 10.1038/nprot.2017.147 29446774

[33] Varnaitė R. and MacNeill S.A.. (2016). Meet the Neighbors: Mapping Local Protein Interactomes by Proximity-Dependent Labeling with Bioid. Proteomics, 16(19), 2503–2518. 27329485 10.1002/pmic.201600123 PMC5053326

[34] Kim D.I., Cutler J.A., Na C.H., Reckel S., Renuse S., Madugundu A.K., Tahir R., Goldschmidt H.L., Reddy K.L., Huganir R.L., Wu X., Zachara N.E., Hantschel O., and Pandey A.. (2018). Biosite: A Method for Direct Detection and Quantitation of Site-Specific Biotinylation. J Proteome Res, 17(2), 759–769. 10.1021/acs.jproteome.7b00775 29249144 PMC6092923

[35] Chung C.Y., Khurana V., Yi S., Sahni N., Loh K.H., Auluck P.K., Baru V., Udeshi N.D., Freyzon Y., Carr S.A., Hill D.E., Vidal M., Ting A.Y., and Lindquist S.. (2017). In Situ Peroxidase Labeling and Mass-Spectrometry Connects Alpha-Synuclein Directly to Endocytic Trafficking and Mrna Metabolism in Neurons. Cell Syst, 4(2), 242–250. e4. 10.1016/j.cels.2017.01.002 28131823 PMC5578869

[36] Wang L., Das U., Scott D.A., Tang Y., McLean P.J., and Roy S.. (2014). α-Synuclein Multimers Cluster Synaptic Vesicles and Attenuate Recycling. Current Biology, 24(19), 2319–2326. 25264250 10.1016/j.cub.2014.08.027 PMC4190006

[37] Dettmer U., Ramalingam N., von Saucken V.E., Kim T.E., Newman A.J., Terry-Kantor E., Nuber S., Ericsson M., Fanning S., Bartels T., Lindquist S., Levy O.A., and Selkoe D.. (2017). Loss of Native α-Synuclein Multimerization by Strategically Mutating Its Amphipathic Helix Causes Abnormal Vesicle Interactions in Neuronal Cells. Human Molecular Genetics, 26(18), 3466–3481. 10.1093/hmg/ddx227 28911198 PMC5884392

[38] Burré J., Sharma M., and Südhof T.C.. (2014). α-Synuclein Assembles into Higher-Order Multimers Upon Membrane Binding to Promote Snare Complex Formation. Proceedings of the National Academy of Sciences, 111(40), E4274–83. 10.1073/pnas.1416598111 PMC4210039 25246573

[39] Koopman M.B., Ferrari L., and Rüdiger S.G.. (2022). How Do Protein Aggregates Escape Quality Control in Neurodegeneration? Trends in Neurosciences, 45(4), 257–271. 10.1016/j.tins.2022.01.006 35210101

[40] Parra-Rivas L.A., Madhivanan K., Aulston B.D., Wang L., Prakashchand D.D., Boyer N.P., Saia-Cereda V.M., Branes-Guerrero K., Pizzo D.P., Bagchi P., Sundar V.S., Tang Y., Das U., Scott D.A., Rangamani P., Ogawa Y., and Subhojit R.. (2023). Serine-129 Phosphorylation of α-Synuclein Is an Activity-Dependent Trigger for Physiologic Protein-Protein Interactions and Synaptic Function. Neuron, 111(24), 4006–4023.e10. 10.1016/j.neuron.2023.11.020 38128479 PMC10766085

[41] Won S.Y., Park M.H., You S.T., Choi S.W., Kim H.K., McLean C., Bae S.C., Kim S.R., Jin B.K., Lee K.H., Shin E.Y., and Kim E.G.. (2016). Nigral Dopaminergic Pak4 Prevents Neurodegeneration in Rat Models of Parkinson’s Disease. Science Translational Medicine, 8(367), 367ra170. 10.1126/scitranslmed.aaf1629 27903866

[42] Won S.-Y., Park J.-J., You S.-T., Hyeun J.-A., Kim H.-K., Jin B.K., McLean C., Shin E.-Y., and Kim E.-G.. (2022). P21-Activated Kinase 4 Controls the Aggregation of α-Synuclein by Reducing the Monomeric and Aggregated Forms of α-Synuclein: Involvement of the E3 Ubiquitin Ligase Nedd4–1. Cell Death Dis, 13(6), 575. 10.1038/s41419-022-05030-1 35773260 PMC9247077

[43] Cappelletti C., Henriksen S.P., Geut H., Rozemuller A.J., van de Berg W.D., Pihlstrøm L., and Toft M.. (2023). Transcriptomic Profiling of Parkinson’s Disease Brains Reveals Disease Stage Specific Gene Expression Changes. Acta Neuropathologica, 146(2), 227–244. 10.1007/s00401-023-02597-7 37347276 PMC10329075

[44] Jian X., Zhao G., Chen H., Wang Y., Li J., Xie L., and Li B.. (2022). Revealing a Novel Contributing Landscape of Ferroptosis-Related Genes in Parkinson’s Disease. Comput Struct Biotechnol J, 20, 5218–5225. 10.1016/j.csbj.2022.09.018 36187920 PMC9508518

[45] Bassil F., Delamarre A., Canron M.H., Dutheil N., Vital A., Négrier-Leibreich M.L., Bezard E., Fernagut P.O., and Meissner W.G.. (2022). Impaired Brain Insulin Signalling in Parkinson’s Disease. Neuropathol Appl Neurobiol, 48(1), e12760. 10.1111/nan.12760 34405431

[46] Aviles-Olmos I., Limousin P., Lees A., and Foltynie T.. (2013). Parkinson’s Disease, Insulin Resistance and Novel Agents of Neuroprotection. Brain, 136(Pt 2), 374–84. 10.1093/brain/aws009 22344583

[47] Athauda D. and Foltynie T.. (2016). Insulin Resistance and Parkinson’s Disease: A New Target for Disease Modification? Progress in neurobiology, 145, 98–120. 10.1016/j.pneurobio.2016.10.001 27713036

[48] Kalinderi K., Papaliagkas V., and Fidani L.. (2024). Glp-1 Receptor Agonists: A New Treatment in Parkinson’s Disease. Int J Mol Sci, 25(7). 10.3390/ijms25073812 PMC11011817 38612620

[49] Benarroch E. (2025). What Is the Function and Relevance of 14–3-3 Proteins in Neurologic Disease? Neurology, 104(5), e213418. 10.1212/wnl.0000000000213418 39889260

[50] Underwood R., Gannon M., Pathak A., Kapa N., Chandra S., Klop A., and Yacoubian T.A.. (2021). 14–3-3 Mitigates Alpha-Synuclein Aggregation and Toxicity in the in Vivo Preformed Fibril Model. Acta Neuropathologica Communications, 9(1), 13. 10.1186/s40478-020-01110-5 33413679 PMC7792107

[51] Hallacli E., Kayatekin C., Nazeen S., Wang X.H., Sheinkopf Z., Sathyakumar S., Sarkar S., Jiang X., Dong X., and Di Maio R.. (2022). The Parkinson’s Disease Protein Alpha-Synuclein Is a Modulator of Processing Bodies and Mrna Stability. Cell, 185(12), 2035–2056. e33. 35688132 10.1016/j.cell.2022.05.008 PMC9394447

[52] Belur N.R., Bustos B.I., Lubbe S.J., and Mazzulli J.R.. (2024). Nuclear Aggregates of Nono/Sfpq and a-to-I-Edited Rna in Parkinson’s Disease and Dementia with Lewy Bodies. Neuron, 112(15), 2558–2580.e13. 10.1016/j.neuron.2024.05.003 38761794 PMC11309915

